# Supplementary material for: A metabolome atlas of the aging mouse brain
Source: Nat Commun. 2021 Oct 15;12:6021. doi: 10.1038/s41467-021-26310-y (PMC8519999; doi:10.1038/s41467-021-26310-y)
Supplement: Supplementary file 1 — Supplementary Information [file 41467_2021_26310_MOESM1_ESM.pdf]

Supplementary Information for:

A Metabolome Atlas of the Aging Mouse Brain

Jun Ding[1,2], Jian Ji[3], Zachary Rabow[1], Tong Shen[1], Jacob Folz[1], Christopher R. Brydges[1], Sili Fan[1], Xinchen Lu[1], Sajjan Mehta[1], Megan R. Showalter[1], Ying Zhang[1], Renee Araiza[4], Lynette R Bower[4], K. C. Kent Lloyd[4], Oliver Fiehn[1,*]

1. West Coast Metabolomics Center, UC Davis Genome Center, University of California, Davis, 451 Health Sciences Drive, Davis, California 95616, United States

2. Department of Chemistry, Wuhan University, Wuhan, Hubei 430072, PR China

3. School of Food Science, State Key Laboratory of Food Science and Technology, National Engineering Research Center for Functional Foods, Synergetic Innovation Center of Food Safety and Nutrition, Jiangnan University, Wuxi, Jiangsu 214122, PR China

4. Mouse Biology Program, and Department of Surgery, School of Medicine, University of California-Davis, Davis, CA, 95618, USA

(*) Corresponding author Email

ofiehn@ucdavis.edu

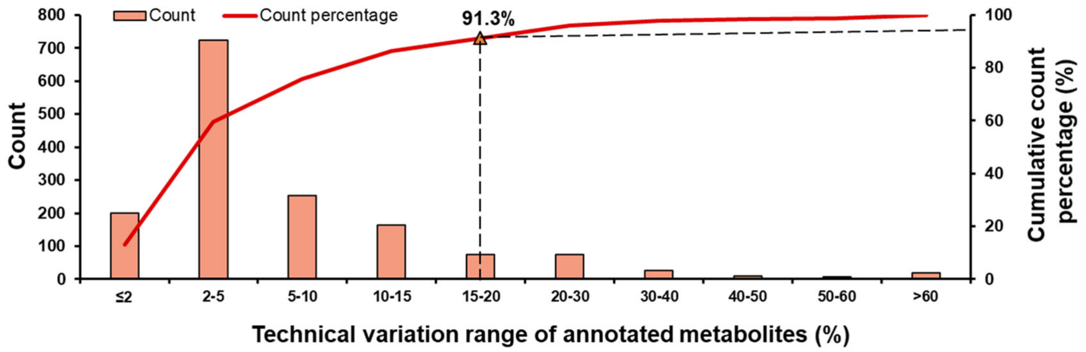

Supplementary Figure 1. Distribution and cumulative percentage of technical variation of annotated metabolites in QC samples.

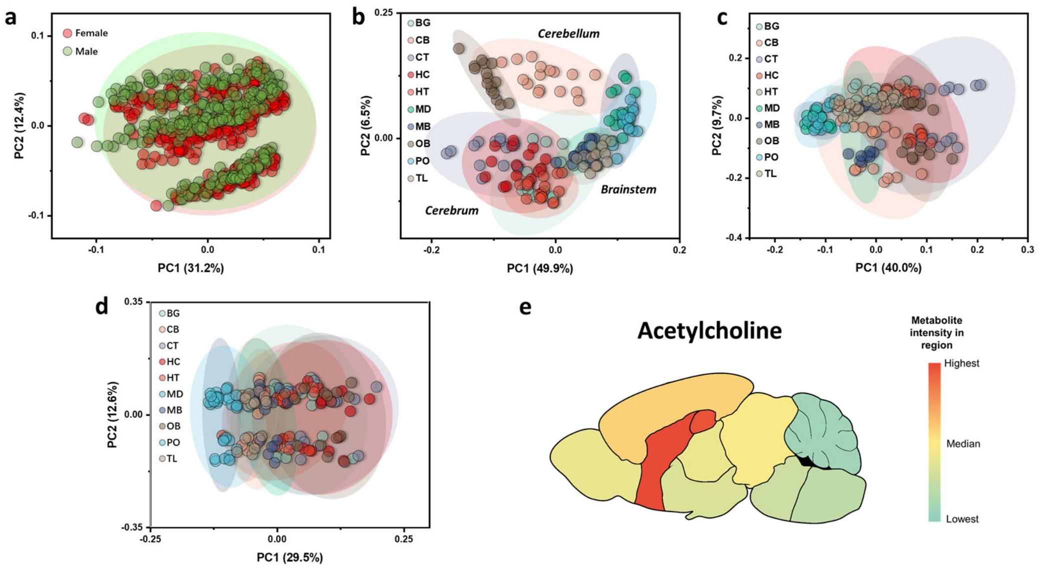

Supplementary Figure 2. Analysis of brain metabolome architecture.

a. Multivariate analysis of mouse brain metabolome dataset via PCA shows there is minor gender difference. Samples are colored based on gender.
b. Principal Component Analysis (PCA) focused on adolescent mice for all 10 brain regions. Samples are colored by brain regions.
c. Principal Component Analysis (PCA) focused on middle-aged mice for all 10 brain regions. Samples are colored by brain regions.
d. Principal Component Analysis (PCA) focused on old-aged mice for all 10 brain regions. Samples are colored by brain regions.
e. The distribution of acetylcholine in the adolescent mouse brain.

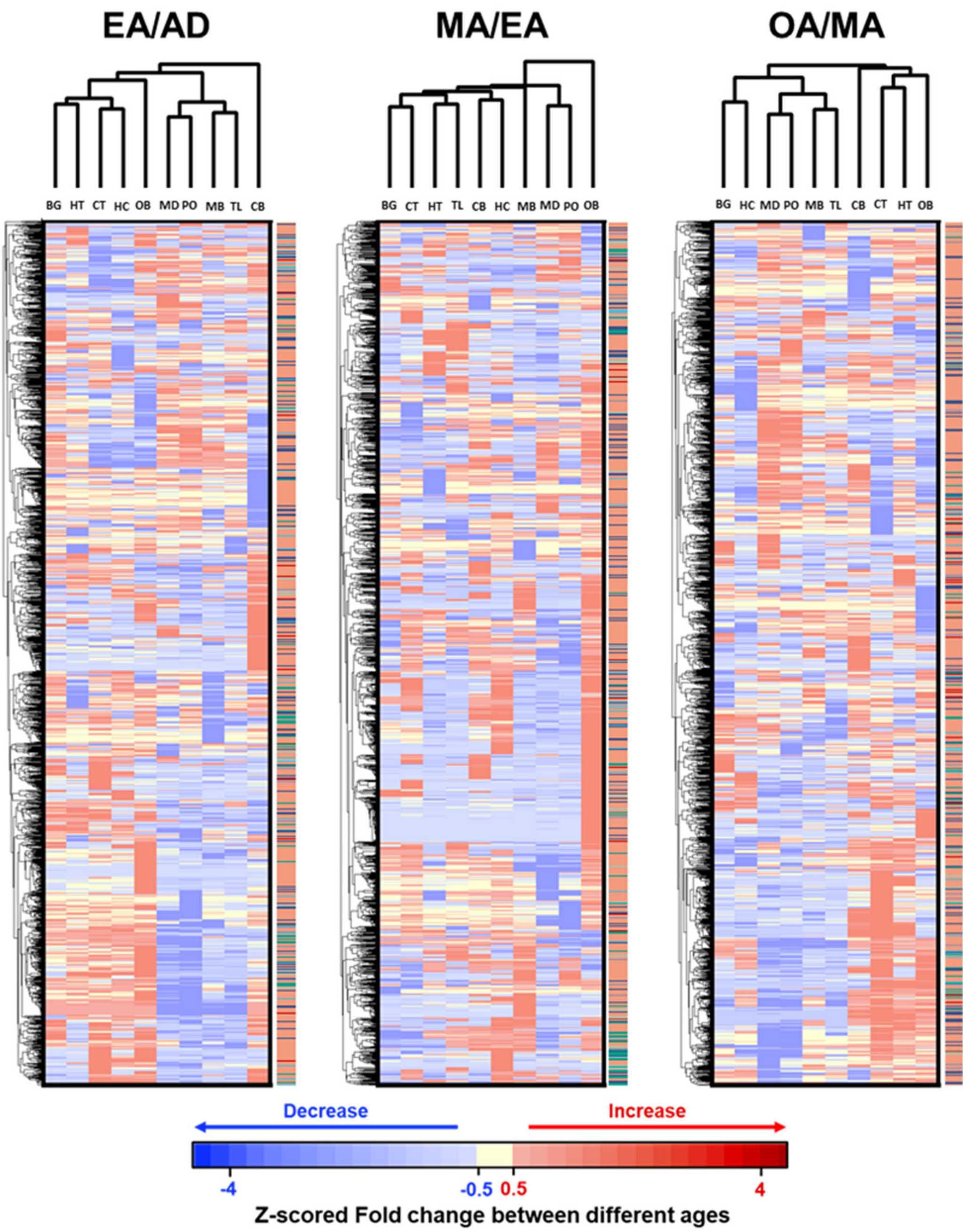

Supplementary Figure 3. Heatmap of altered metabolites over aging. The heat map is based on the z-scored fold changes of early adults/adolescents (left), middle ages/early adults (middle), and old ages/middle ages (right) after unsupervised hierarchical clustering. Metabolite classes are colored by ClassyFire categories. Benzenoids: red, Lipids: orange, Nucleosides: light green, Acids: dark blue, Nitrogen organics: purple, Oxygen organics: dark green, Heterocyclics: light blue, Others: dark grey.
