# Supplementary material for: A metabolome atlas of the aging mouse brain
Source: Nat Commun. 2021 Oct 15;12:6021. doi: 10.1038/s41467-021-26310-y (PMC8519999; doi:10.1038/s41467-021-26310-y)
Supplement: Supplementary file 3 — Description of Additional Supplementary Files [file 41467_2021_26310_MOESM3_ESM.docx]

**Description of Additional Supplementary Files**

File Name: Supplementary Data 1

Description: Basic information of the mice.

File Name: Supplementary Data 2.

Description: Mouse brain atlas dataset.

File Name: Supplementary Data 3.

Description: Quantification results of mouse brain metabolites.

File Name: Supplementary Data 4.

Description: Pathway-based sets informed by Consensus PathDB.

File Name: Supplementary Data 5.

Description: Regional specific metabolites.
