# Supplementary material for: A metabolome atlas of the aging mouse brain
Source: Nat Commun. 2021 Oct 15;12:6021. doi: 10.1038/s41467-021-26310-y (PMC8519999; doi:10.1038/s41467-021-26310-y)
Supplement: Supplementary file 9 — Reporting Summary [file 41467_2021_26310_MOESM9_ESM.pdf]

nature portfolio

Corresponding author(s): Fiehn

Last updated by author(s): Sep 16, 2021

Reporting Summary

Nature Portfolio wishes to improve the reproducibility of the work that we publish. This form provides structure for consistency and transparency in reporting. For further information on Nature Portfolio policies, see our Editorial Policies and the Editorial Policy Checklist.

Statistics

For all statistical analyses, confirm that the following items are present in the figure legend, table legend, main text, or Methods section.

n/a	Confirmed	
☐	☒	The exact sample size (n) for each experimental group/condition, given as a discrete number and unit of measurement
☐	☒	A statement on whether measurements were taken from distinct samples or whether the same sample was measured repeatedly
☐	☒	The statistical test(s) used AND whether they are one- or two-sided<br>*Only common tests should be described solely by name; describe more complex techniques in the Methods section.*
☐	☒	A description of all covariates tested
☐	☒	A description of any assumptions or corrections, such as tests of normality and adjustment for multiple comparisons
☐	☒	A full description of the statistical parameters including central tendency (e.g. means) or other basic estimates (e.g. regression coefficient) AND variation (e.g. standard deviation) or associated estimates of uncertainty (e.g. confidence intervals)
☐	☒	For null hypothesis testing, the test statistic (e.g. F, t, r) with confidence intervals, effect sizes, degrees of freedom and P value noted<br>*Give P values as exact values whenever suitable.*
☒	☐	For Bayesian analysis, information on the choice of priors and Markov chain Monte Carlo settings
☐	☒	For hierarchical and complex designs, identification of the appropriate level for tests and full reporting of outcomes
☐	☒	Estimates of effect sizes (e.g. Cohen's d, Pearson's r), indicating how they were calculated

Our web collection on statistics for biologists contains articles on many of the points above.

Software and code

Policy information about availability of computer code

Data collection	LC-MS/MS data was acquired on a ThermoFisher Q-Exactive HF mass spectrometer by the commercial Xcalibur™ software from Thermo Fisher Scientific. GC-MS data was obtained by a Leco Pegasus IV time-of-flight mass spectrometer with the ChromaTOF software version 4.50.
Data analysis	For GC-MS data analysis, ChromaTOF version 4.50 was employed for baseline subtraction, deconvolution and peak detection, and Binbase was used for metabolite annotation and reporting. Open source MS-DIAL ver. 4.00 software was used for the deconvolution, peak picking, alignment, and compound identification of LC-MS/MS data. Open source MS-FLO software at https://msflo.fiehnlab.ucdavis.edu/ was used to identify ion adducts, duplicate peaks, and isotopic features. Open source SERRF software at https://slfan2013.github.io/SERRF-online/ was employed to correct for batch effects or instrument signal drifts.

For manuscripts utilizing custom algorithms or software that are central to the research but not yet described in published literature, software must be made available to editors and reviewers. We strongly encourage code deposition in a community repository (e.g. GitHub). See the Nature Portfolio guidelines for submitting code & software for further information.

Data

Policy information about availability of data

All manuscripts must include a data availability statement. This statement should provide the following information, where applicable:

- Accession codes, unique identifiers, or web links for publicly available datasets
- A description of any restrictions on data availability
- For clinical datasets or third party data, please ensure that the statement adheres to our policy

This data is available at the NIH Common Fund's National Metabolomics Data Repository (NMDR) website, the Metabolomics Workbench [https://www.metabolomicsworkbench.org] where it has been assigned Project ID (ST001637PR001047). The data can be accessed directly via it's Project DOI: (doi:

10.21228/M8C68D). In situ hybridization images are taken from 2004 Allen Institute for Brain Science. (Allen Human Brain Atlas. Available from: http://mouse.brain-map.org) Image credit: Allen Institute.

Field-specific reporting

Please select the one below that is the best fit for your research. If you are not sure, read the appropriate sections before making your selection.

- [x] Life sciences
- [ ] Behavioural & social sciences
- [ ] Ecological, evolutionary & environmental sciences

For a reference copy of the document with all sections, see nature.com/documents/nr-reporting-summary-flat.pdf

Life sciences study design

All studies must disclose on these points even when the disclosure is negative.

Sample size	The manuscript is focused on studying the metabolome of the aging wildtype male and female mouse brain from 10 anatomical regions spanning from adolescence to old age (four age groups). There were eight biological replicates in each group. And 640 brain samples total were analyzed by three metabolomic assays, including GC-TOF MS, HILIC-MS/MS and lipidomic LC-MS/MS.
Data exclusions	From the total 640 biological samples, three outlier samples were removed by outlier analysis in PCA plots, including one medulla sample from a female early adult,one basal ganglia sample from a middle-aged female, and one olfactory bulb from an old-aged male. For metabolites that were detected by two or more platforms, values with the lowest relative standard deviation in quality control samples were kept. Metabolites that were present in at least 6 of the 8 samples in at least one of the 80 study groups (defined by age, sex and brain region) were kept in the dataset, otherwise metabolites were removed from the dataset. Missing data were replaced by 1/10th of the minimum value (default value 100).
Replication	Pooled quality control samples were used after each set of 10 samples during data acquisition. Identical pools were used for the first set of data acquisitions, and the subsequent additional data acquisitions for old mice. The technical reproducibility of the data was verified by the tight cluster of all pooled QC samples in the PCA plot in Figure 2A, and the low standard deviations of metabolite peak heights in pooled QC samples. The reproducibility of metabolites of biological replicates were verified by Spearman-rank correlation analysis of the samples from the different brain regions.
Randomization	Samples were completely randomized between ages, sexes and brain regions for adolescent-adult mice, and then again for the second (new) dataset on 92-week old mice.
Blinding	Samples were blinded to operators of the mass spectrometers.

Reporting for specific materials, systems and methods

We require information from authors about some types of materials, experimental systems and methods used in many studies. Here, indicate whether each material, system or method listed is relevant to your study. If you are not sure if a list item applies to your research, read the appropriate section before selecting a response.

Materials & experimental systems

n/a	Involved in the study	
x		Antibodies
x		Eukaryotic cell lines
x		Palaeontology and archaeology
	x	Animals and other organisms
x		Human research participants
x		Clinical data
x		Dual use research of concern

Methods

n/a	Involved in the study	
x		ChIP-seq
x		Flow cytometry
x		MRI-based neuroimaging

Animals and other organisms

Policy information about studies involving animals; ARRIVE guidelines recommended for reporting animal research

Laboratory animals	3 weeks, 16 weeks, 59 weeks and 92 weeks old male and female wild type mice on a C57BL/6N background mice were employed in the study. Mice were cohoused by gender groups of 4-5 in individually ventilated cages (Optimice IVC, Animal Care Systems, Centennial, CO) on a 12:12-hour (6:00/18:00) light:dark cycle at 68-79°F with 40-60% humidity and provided water and standard rodent chow (Rodent chow, Harlan 2918) ad libitum.
Wild animals	No wild animals were used in the study.
Field-collected samples	No field collected samples were used in the study.

Ethics oversight	All procedures were approved by the IACUC of the University of California, Davis, which is an AAALAC-accredited institution. Animal housing and euthanasia was performed in accordance with recommendations of the Guide for the Care and Use of Laboratory Animals.

Note that full information on the approval of the study protocol must also be provided in the manuscript.
